# Tremors of the Earth

**Published:** 1882-11

**Authors:** 


### TREMORS OF THE EARTH.

The London *Times* publishes a synopsis of some papers on the "tremors of the earth," by the committee appointed to measure the lunar disturbance of gravity, and by Mr. G. Darwin, which contains some statements new to the public. It is considered proved by thə men of science engaged that the crust of the earth bends under the weights imposed on it, till " when the barometer rises an inch over a land area like that of Australia, the increased load of air sinks the entire continent two or three inches below the normal evel." The land actually sinks and rises under the pressure of the mass of water thrown upon it by the tides, the maximum of rise and fall on the Atlantic seaboard reaching five inches. This effect is felt at the bottom of the deepest mine, and may reach for an unknown distance. It follows that the crust of the earth must be of exceeding tenacity, exceeding as a minimum that of granite; and its swayings may be the causes of phenomena hitherto quite unexplained, as, for example, the relation between storm and earthquake. So universal, frequent and unavoidable are these disturbances that the inquiry into the lunar disturbances of gravity has been given up. No depth can be found at which a recording instrument can be placed so as to escape their effect. The round earth pants, in fact, like a breathing being, under the changes always going on above her.

